# Protective Effects of Otophylloside N on Pentylenetetrazol-Induced Neuronal Injury *In vitro* and *In vivo*

**DOI:** 10.3389/fphar.2016.00224

**Published:** 2016-07-25

**Authors:** Feiya Sheng, Mengting Chen, Yuan Tan, Cheng Xiang, Mi Zhang, Baocai Li, Huanxing Su, Chengwei He, Jianbo Wan, Peng Li

**Affiliations:** ^1^State Key Laboratory of Quality Research in Chinese Medicine, Institute of Chinese Medical Sciences, University of MacauMacau, China; ^2^Faculty of Life Science and Technology, Kunming University of Science and TechnologyKunming, China

**Keywords:** epilepsy, pentylenetetrazol, otophylloside N, *Cynanchum otophyllum* Schneid, neuroprotective effect, apoptosis

## Abstract

Approximately 30% of epileptic patients worldwide are medically unable to control their seizures. In addition, repeated epileptic seizures generally lead to neural damage. Pentylenetetrazol (PTZ) is a clinical circulatory and respiratory stimulant that is experimentally used to mimic epileptic convulsion in epilepsy research. Here, we systematically explore the neuroprotective effects of a pure compound isolated from *Cynanchum otophyllum* Schneid (Qingyangshen), Otophylloside N (OtoN), against PTZ-induced neuronal injury. We used three models: *in vitro* primary cortical neurons, *in vivo* mice, and *in vivo* zebrafish. Our results revealed that OtoN treatment may attenuate PTZ-induced morphology changes, cell death, LDH efflux in embryonic neuronal cells of C57BL/6J mice, and convulsive behavior in zebrafish. Additionally, our Western blot and RT-PCR results demonstrated that OtoN may attenuate PTZ-induced apoptosis and neuronal activation in neuronal cells, mice, and zebrafish. OtoN may reduce PTZ-induced cleavage of poly ADP-ribose polymerase and upregulation of the Bax/Bcl-2 ratio and decrease the expression level of *c-Fos*. This study is the first investigation of the neuroprotective effects of OtoN, which might be developed as a novel antiepileptic drug.

## Introduction

Epilepsy consists of a diverse set of neurological conditions, and it is characterized predominantly by repeated epileptic seizures (Fisher et al., 2005; Löscher et al., 2013; Khoshnoud et al., 2015). Epileptic seizures are transient symptoms that result from excessive abnormal or synchronous cortical nerve cell activities in the brain. Although over 15 third-generation antiepileptic drugs (AEDs) have been introduced to treat epilepsy, approximate 30% of patients are still medically unable to control their seizures (Löscher et al., 2013; Yu et al., 2015). Moreover, approximately 30–40% of all epileptic patients are still affected by substantial side effects and seizure resistance to the currently available AEDs (Schmidt, 2002). On the one hand, many investigations have demonstrated that repetitive and prolonged seizures generally lead to widespread neuronal death (Sankar et al., 1998). Substantial neuronal death is first likely to appear in areas of high susceptibility such as the cortex and hippocampus before spreading widely to other structures. On the other hand, seizure-induced neuronal injury might promote epileptogenesis (Dingledine et al., 2014).

Owing to the rich source and enormous structural diversity, natural compounds such as baicalin (de Carvalho et al., 2011; Liu et al., 2012), ganoderma lucidum polysaccharides (Zhou et al., 2015), saikosaponin (Yu et al., 2012), and berberine (Zhu and Qian, 2006; Bhutada et al., 2010) have been associated with increased concern in recent years. In particular, the effective dosages, the potential mechanisms underlying the anticonvulsant or antiepileptic effects and the structure-activity relationships of some of these active components have been systematically reviewed (Zhu et al., 2014). *Cynanchum otophyllum* Schneid (Chinese name: Qingyangshen) is a widely used herbal medicine to treat epilepsy, lumbar muscle strain, and rheumatism in southwestern China. Modern pharmacological studies have revealed that the total extract of Qingyangshen exhibits remarkable antiepileptic effects (Guo and Kuang, 1996; Li et al., 2006). However, the pure compound responsible for its therapeutic effect still remains unclear, except for otophylloside A and B, which were reported nearly 30 years ago (Mu et al., 1986). Therefore, the discovery of novel constituents from *C. otophyllum* that exhibit antiepileptic activity and neuroprotective effects is very important for further development and utilization of this potential medicinal plant resource.

Pentylenetetrazol (PTZ) is a circulatory and respiratory stimulant that is clinically used to treat respiratory depression and acute circulatory failure caused by drug (e.g., narcotic) poisoning. However, high dosages of PTZ may result in convulsions, which share similar behavioral and are pharmacological character with human absence seizures, and this type of convulsive actions can strongly mimic epileptic seizures. Therefore, PTZ has been experimentally subjected as a model inducer in the exploration of AEDs (Vlainić and Pericić, 2009; Löscher et al., 2013). In this study, we evaluated Otophylloside N (OtoN), a C21 steroidal glycoside isolated from *C. otophyllum*, for its neuroprotective effects. We used three models, including primary cortical neurons, mice and zebrafish, to systematically investigate the protective effects of OtoN against PTZ-induced neuronal injury both *in vitro* and *in vivo*.

## Materials and Methods

### Animal Ethics Statement and Maintenance

The Animal Ethics Committee of the Institute of Chinese Medical Sciences, University of Macau approved this study (Ethical NO. ICMS-AEC-2015-20). All of the procedures performed on the animals were carried out in strict accordance with the ethical guidelines of Institute of Chinese Medical Sciences, University of Macau.

C57BL/6J mice aged 8–10 weeks were housed in standard cages (temperature: 21 ± 1°C; humidity: 60 ± 5%) with free access to food and water. The animal room was subjected to be 12-hour light/12-hour dark cycle (lights on at 08:00). All the mice were euthanized by rapidly cervical dislocation.

We maintained wild-type zebrafish according to the standard procedures described in the Zebrafish Handbook (Westerfield, 1995). The stocks were maintained at a temperature of 28.5 ± 1°C in a 14-hour light/10-hour dark cycle in embryo medium (13.7 mM NaCl, 540 μM KCl, 25 μM Na_2_HPO_4_, 44 μM KH_2_PO_4_, 300 μM CaCl_2_, 100 μM MgSO_4_, 420 μM NaHCO_3_, pH 7.4). The zebrafish were euthanized with overdose of MS-222 (3-Aminobenzoic acid ethyl ester methanesulfonate) after locomotor behavioral monitoring.

### Preparation of Primary Cortical Neurons

We obtained primary cortical neurons from E16 mice embryos. Briefly, the cortical tissue was dissected from embryonic brain tissue in ice-cold DMEM/F12 medium and digested in 0.05% trypsin for 15 min at 37°C. The cell density was adjusted to 5 × 10^5^/mL. Next, the cells were planted in poly-L-lysine and Laminin (Sigma–Aldrich, St. Louis, MO, USA) coated culturing ware in neurobasal medium with 2% B-27 supplement, 0.5% GlutaMAX-I^TM^ supplement, and 1% penicillin-streptomycin (Gibco, USA). The culture medium was half replaced every 3 days.

### Drug Administration

**FIGURE 1:**
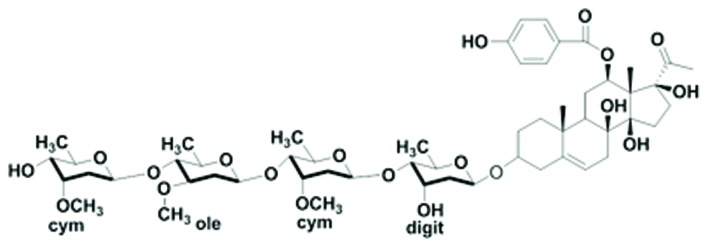
**Chemical structure of Otophylloside N**.

Pentylenetetrazol was purchased from Sigma–Aldrich and freshly prepared prior to use. OtoN was isolated from *C. otophyllum* and identified by the Faculty of Life Science and Technology, Kunming University of Science and Technology (Kunming, China); its purity exceeded 98% (**Figure 1**).

We cultured the primary cells for 7 days *in vitro* (DIV) prior to treating them with isometric fresh medium, PTZ (30 mM), and PTZ (30 mM) + OtoN of different dosages.

The mice were injected intraperitoneally with phosphate buffer solution (PBS), PTZ (30 mg/kg), and PTZ (30 mg/kg) + OtoN of different dosages in corresponding groups. These injections were conducted between 09:00 and 10:00 every morning and repeated for 7 days.

Zebrafish larvae at 6 days post fertilization (dpf) were pretreated with fresh embryo medium with different dosages of OtoN for 24 h and then moved into medium or 10 mM PTZ diluted in medium.

### Immunocytochemistry

The drug-treated neurons were fixed with 4% paraformaldehyde (Sigma–Aldrich) at room temperature. After three washes with PBS, the cells were permeabilized with blocking buffer (0.3% Triton X-100, 3% BSA, and 10% goat serum in PBS). The cells were then incubated with primary antibodies, including anti-MAP2 antibody (Novex, San Diego, CA, 1:500) or anti-β-Tubulin antibody (Invitrogen, CA, 1:500) and anti-GFAP antibody (Abcam, USA, 1:1000) at 4°C overnight, and followed with corresponding secondary antibodies for 1 h at room temperature in the dark. Finally, the cell nuclei were counterstained with 5 μg/ml of DAPI (Sigma–Aldrich) for 15 min. The samples were photographed using an InCell2000 Analyzer (GE Healthcare, USA).

### CCK-8 Assay

Cell Counting Kit reagents (Beyotime, Shanghai, China) were added to the culturing medium (1:10) in each well of a 96-well plate. After incubating the mixture for 4 h at 37°C, the absorbance was measured using a FlexStation III microplate reader (Molecular Devices, USA) at a wavelength of 450 nm.

### Lactate Dehydrogenase Activity Assessment

LDH enzyme activity was assayed using a Cytotoxicity Detection Kit (Roche Mannheim, Germany). 100 μL of the reaction mixture of the kit was added to the treated neurons in a 96-well plate followed by incubation at 37°C for 30 min in a dark environment. The absorbance was determined using a microplate reader (Molecular Devices) at a wavelength of 490 nm.

### Western Blot Analysis

The cells were lysed in RIPA lysis buffer containing 1% protease inhibitor cocktail and 1% phenylmethanesulfonyl fluoride. For the *in vivo* study, the cerebral cortex tissues were gently dissected from the drug-treated mice and homogenized to suspension, and then followed the same lysis step as described above for the cells. We determined the protein concentration of the lysates using a BCATM Protein Assay Kit (Pierce, Rockford, IL, USA). The proteins were separated using SDS-PAGE and then transferred to polyvinylidene fluoride membranes, blocked with 5% non-fat milk for 2 h at room temperature. The immunoreactions were carried out using poly ADP-ribose polymerase (PARP), Bax and Bcl-2 antibodies (Abcam, 1:1000), and then the membranes were probed with the corresponding secondary antibodies for 1 h at room temperature. Specific protein bands were visualized using the ECL Select Western Blotting Detection Reagent (GE Healthcare, Buckinghamshire, UK). Equal protein loading was verified by probing with anti-GAPDH antibodies.

### Locomotor Behavioral Monitoring of Zebrafish

The zebrafish larvae were placed individually into 96-well microplates with 12 larvae in each group and habituated to their new environment for 30 min. The larvae were then treated with embryo medium or 10 mM PTZ in each well. After being pretreated with the indicated compound for 15 min in an incubator (28.5 ± 1°C), the plates were housed inside the Zebrabox and monitored using a ZebraLab video tracking system (Viewpoint Life Science, France). We recorded the swimming behaviors of the larvae for 30 min. In the pre-experiments, we defined three different velocity ranges: inaction (0–4 mm/s), small movement (4–20 mm/s), and large movement (above 20 mm/s).

### Reverse Transcriptase-Polymerase Chain Reaction

Approximately 55 zebrafish larvae at 7 dpf in each group were homogenized for 5 min. Then, the total RNA was extracted from the tissues using the RNeasy Plus Mini Kit (Qiagen, Germany) in accordance with the manufacturer’s instructions. The RNA concentration and purity were measured using NanoVue Plus ROW (GE Healthcare). Then, the total RNA was reverse-transcribed to single-strand cDNA using a PrimeScript RT Reagent Kit (Perfect Real Time, Japan). Next, we carried out quantitative real-time (RT) PCR on a ViiA 7 RT PCR System (Life Technologies, Carlsbad, CA, USA) according to the SYBR assay. The following primer sequences were selected (Marques et al., 2008; Deng et al., 2009; Santos et al., 2014; Vanhauwaert et al., 2014) : zebrafish β-actin, ACGATGGATGGGAAGACA and AAATTGCCGCACTGGTT; zebrafish *c-Fos*, TTACCCGCTCAACCAGACTC and TGACAGTTGGCACGAAAGAG; zebrafish Bcl-2, TCACTCAGTTCAGACCCTCAT and ACGCTTTCCACGCACAT; zebrafish Bax, TCACTCAGTTCAGACCCTCAT and ACGCTTTCCACGCACAT. All of the oligonucleotide primers were synthesized by Invitrogen. Expressions of these genes were normalized to β-actin using the relative quantification method.

### Statistical Analysis

Data were expressed as mean ± standard deviation (SD). We analyzed the statistical significances using one-way analysis of variance (ANOVA) with the SPSS 17 software (Statistical Package for the Social Sciences, Chicago, IL, USA). ^∗^/^Δ^*P* < 0.05 and ^∗∗^/^ΔΔ^*P* < 0.01 were regarded as being statistically significant.

## Results

### PTZ-Induced Neurotoxicity on Primary Cortical Neurons

**FIGURE 2:**
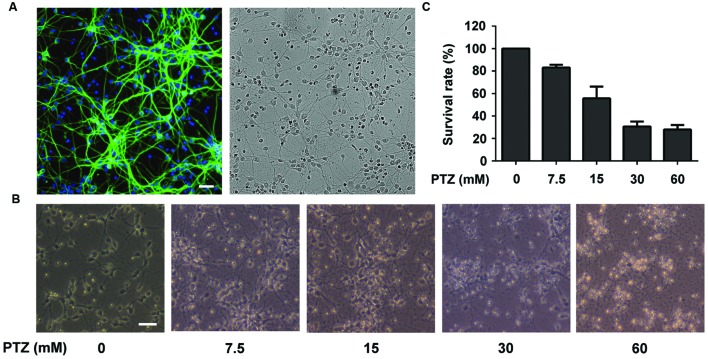
**Investigation of Pentylenetetrazol (PTZ)-induced toxicity in primary cortical neurons. (A)** Immunofluorescence detection of primary neuronal cells and bright field of the same area. Cells were stained with mouse anti-MAP2 antibody (green) and rabbit anti-GFAP antibody (red, detection of astrocytes). The nuclei are stained with DAPI (blue). **(B)** Observation of morphological changes of neurons using a contrast microscope at 7 days *in vitro* (DIV). The cells were treated with 0, 7.5, 15, 30, and 60 mM PTZ for 24 h at 7 DIV. **(C)** PTZ-induced cellular toxicity in neurons. Cell viability is presented as a percentage of control, and each value represents the mean ± SD of three independent experiments. Bar: 100 μm.

Qualifying the purity of neuronal cells is crucial for follow-up research as well as for ensuring the reliability of the results obtained. We first performed the qualification test. In the immunocytochemistry studies, the cortical neurons were stained with anti-MAP2 antibody (green) while the presence of astrocytes was marked by anti-GFAP antibody (red). The nuclei were counter stained with DAPI (blue). As shown in **Figure 2A**, no astrocytes were found, which revealed that the purity of the neuronal cells was high enough (>95%) for our research.

Pentylenetetrazol is a well-demonstrated chemical inducer that mimics epileptic seizures. Exposure to PTZ (24 h) may result in toxicity to neuronal cells, such as an altered morphology, neurotransmission imbalance and cell death (Naseer et al., 2009). As shown in **Figure 2B**, after treatment of PTZ at concentrations of 7.5, 15, 30, and 60 mM, the cell morphologies were damaged in a dose-dependent manner. The cell survival rates were reduced to 83.06 ± 2.61, 55.69 ± 10.48, 30.68 ± 4.41, and 27.99 ± 3.91%, respectively (**Figure 2C**). Compared with cells treated with 7.5 and 15 mM PTZ, the group treated with 30 mM PTZ exhibited more visible injury and a lower cell survival rate. Moreover, we noted that 30 mM PTZ maintained basic cellular shape and yielded a similar survival rate of neurons as in the 60 mM group. Therefore, 30 mM was selected as the dose for inducing neurotoxicity in PTZ-treated neurons.

### OtoN Attenuated PTZ-Induced Cell Injury on Primary Cortical Neurons

**FIGURE 3:**
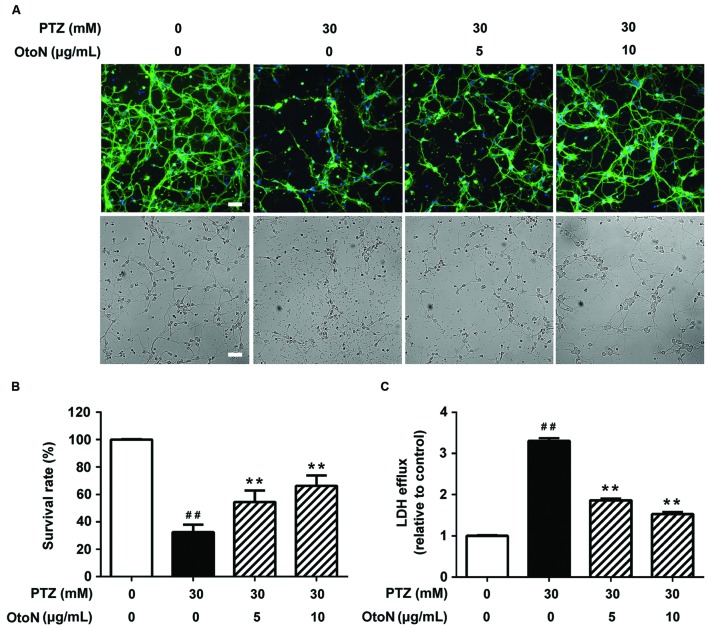
**Otophylloside N attenuated PTZ-induced cell injury on primary cortical neurons.** The neurons were treated with indicated concentrations of PTZ or PTZ + Otophylloside N (OtoN) for 24 h at 7 DIV. **(A)** Immunofluorescence detection of morphological changes of neurons and bright field of the same area. The cells were immunostained with neuronal anti-β-Tubulin marker (green) and nuclear DAPI tag (blue). **(B)** OtoN inhibited PTZ-induced toxicity in neurons. Cell viability is presented as a percentage of the control, and each value represents the mean ± SD of three independent experiments. **(C)** OtoN decreased PTZ-induced LDH intracellular efflux in neurons. The LDH efflux level is presented as a percentage of the control, and each value represents the mean ± SD of three independent experiments. ^##^*P* < 0.01 compared with the control. ^∗∗^*P* < 0.01 compared with the 30 mM PTZ treatment. Bar: 100 μm.

In the previous screening of pure compounds from *C. otophyllum*, we found that OtoN could remarkably improve PTZ-induced neurotoxicity. β-Tubulin is a major component of the eukaryotic cytoskeleton, and fluorescence images adequately illustrate the cell morphology. In this study, we immunostained the cells with neuronal β-Tubulin marker (green) and nuclear DAPI tag (blue). As indicated in **Figure 3A**, the majority of neurons in the PTZ group floated, and the existing cells were adherent loosely and became shrinkage. Moreover, the synapses of these cells were extremely injured as lysing in medium. OtoN (5, 10 μg/mL)-involved groups exhibited more neuronal cells that we preserved and denser maintained synapses than the PTZ group.

We conducted CCK-8 assays to evaluate cell viability. After drug administration, the two doses of OtoN attenuated PTZ-induced cell death by increasing the cell survival rates from 32.45 ± 5.53 to 54.41 ± 8.42 and 66.26 ± 7.58%, respectively (**Figure 3B**). In addition, leakage of LDH is a well-demonstrated sign of injury on cellular membrane (Mohamed et al., 2014), and our results revealed that the involvement of OtoN (5, 10 μg/mL) obviously decreased the intracellular LDH efflux level (1.86 ± 0.04, 1.53 ± 0.05 fold) caused by PTZ (3.30 ± 0.07 fold) (**Figure 3C**).

### OtoN Decreased PTZ-Induced Cell Apoptosis and Activation Upon Cortical Neurons

**FIGURE 4:**
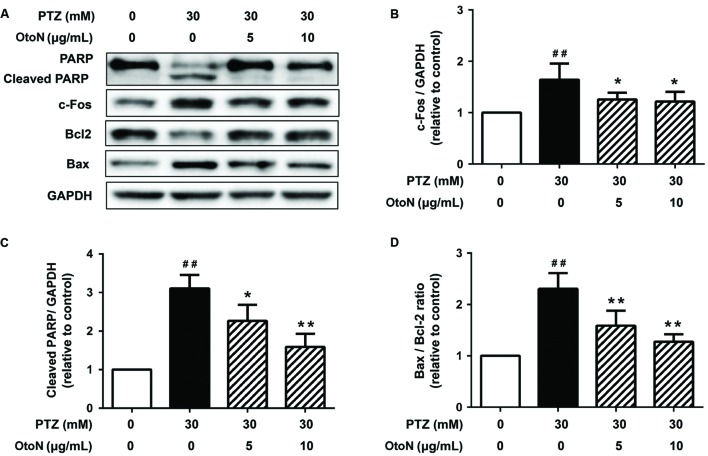
**Otophylloside N decreased PTZ-induced cell apoptosis and neuronal activation upon cortical neurons.** The neurons were treated with isometric fresh medium and the indicated concentrations of PTZ or PTZ + OtoN for 24 h at 7 DIV. **(A)** Protein levels of poly ADP-ribose polymerase (PARP), Bcl-2, Bax, and *c-Fos* were determined by Western blot analysis. **(B–D)** The relative *c-Fos*, cleaved PARP, and Bax/Bcl-2 ratio were calculated. ^##^*P* < 0.01 compared with controls. ^∗^*P* < 0.05 and ^∗∗^*P* < 0.01 compared with the 30 mM PTZ treatment.

Since apoptosis is a common cell-death pathway, we tested the levels of several related proteins to verify whether PTZ induced apoptosis as well as the protective effects of OtoN. Western blot analysis of neuronal cells (**Figure 4A**) revealed that PTZ obviously induced the upregulation of the PARP cleaved fragment 3.10 ± 0.35 fold, indicating the induction of apoptosis. Meanwhile, the Bax/Bcl-2 ratio, an indicator that is generally regarded as determining the susceptibility of cells to the induced apoptosis, was also upregulated 2.31 ± 0.30 fold after treatment with PTZ. With OtoN exposure, the upregulation trends of the two indicators were significantly decreased (**Figures 4C,D**), indicating that OtoN decreased PTZ-induced cell apoptosis. *c-Fos* has been reported to be involved in response to neuronal activation. Our results revealed that *c-Fos* was also activated during PTZ treatment and that OtoN may decrease PTZ-induced cortical neuron activation (**Figure 4B**).

### OtoN Abated PTZ-Induced Cell Apoptosis and Neuronal Activation in Mouse Cerebral Cortex

**FIGURE 5:**
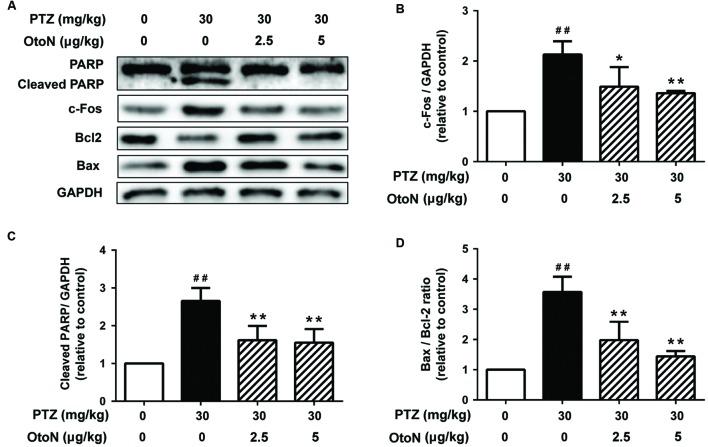
**Otophylloside N abated PTZ-induced cell apoptosis and neuronal activation in mouse cerebral cortex.** The mice were injected intraperitoneally with phosphate buffer solution (PBS), PTZ (30 mg/kg), and PTZ (30 mg/kg) + the indicated concentrations of OtoN in corresponding groups. This injection were conducted between 09:00 and 10:00 every morning and repeated for 7 days. **(A)** Protein levels of PARP, Bcl-2, Bax, and *c-Fos* were determined by Western blot analysis. **(B–D)** We calculated the relative *c-Fos*, cleaved PARP, and Bax/Bcl-2 ratio. ^##^*P* < 0.01 compared with controls. ^∗^*P* < 0.05 and ^∗∗^*P* < 0.01 compared with the 30 mg/kg PTZ treatment.

To confirm the results obtained from the primary neuronal cells, we further detected the expression levels of the proteins noted above in the mouse cerebral cortex *in vivo*. As shown in **Figure 5**, PTZ obviously induced the upregulation of PARP cleavage and Bax/Bcl-2 ratio, indicating the induction of apoptosis in the mouse cerebral cortex. Similarly, OtoN at concentrations of 2.5 and 5 μg/mL obviously abated PTZ-induced upregulation of PARP cleavage and decreased the Bax/Bcl-2 ratio (**Figures 5C,D**). Meanwhile, PTZ-induced *c-Fos* production was also decreased with co-treatment with OtoN (**Figure 5B**). These results are consistent with the outcomes on neurons *in vitro*.

### OtoN Reduced PTZ-Induced Neurotoxicity in Zebrafish

**FIGURE 6:**
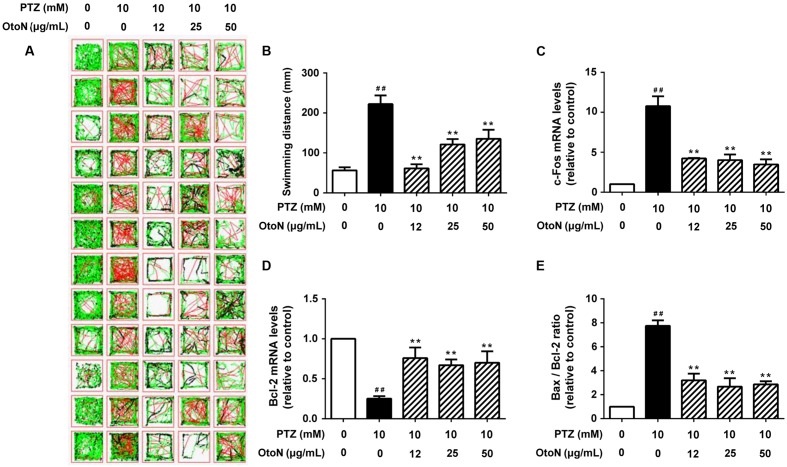
**Otophylloside N reduced PTZ-induced neurotoxicity in zebrafish.** Zebrafish larvae 6 days after fertilization were pretreated with fresh embryo medium and the indicated concentrations of OtoN for 24 h and then changed into fresh medium or 10 mM PTZ diluted in the medium. **(A)** Tracking lines of 30-minute locomotor behaviors of individual larva. The three different colored lines represent different velocity ranges (black = 0–4 mm/s; green = 4–20 mm/s; red = 20+ mm/s). **(B)** We calculated the swimming distances of each group. Each value represents the mean ± SD of three independent experiments. **(C–E)** The relative *c-Fos*, Bcl-2 gene expression levels, and Bax/Bcl-2 ratio was calculated. ^##^*P* < 0.01 compared with controls. ^∗∗^*P* < 0.01 compared with the 30 mM PTZ treatment.

In a locomotor assay, PTZ-treated zebrafish exhibited high-velocity epilepsy seizures that were significantly different from those of the control group. In this case, we presented the results according to large movements, which were visible as a red line in the tracking images. As shown in **Figure 6A**, the activities of the zebrafish in the PTZ group were significantly increased compared to those of the control group, and the OtoN treatment decreased the severe behaviors (the red line) on the contrary. OtoN (12, 25 and 50 μg/mL) reduced the fast swimming distance to 61.43 ± 10.09, 121.1 ± 13.65, 135.5 ± 22.36 mm from 222.2 ± 21.7 mm for the PTZ group (**Figure 6B**).

Beside behavior trial, we next tested gene expression in zebrafish to confirm the prior results of apoptosis and activation. According to the results of RT PCR, the PTZ model exhibited an obvious increase (10.77 ± 1.24 fold) of *c-Fos* compared with the control group (**Figure 6C**). OtoN (12, 25 and 50 μg/mL) pretreatment downregulated the elevated *c-Fos* expression (4.22 ± 0.07, 4.01 ± 0.71, and 3.48 ± 0.63 fold, respectively). In terms of the apoptotic aspect, the ratio of Bax/Bcl-2 was also significantly reduced by OtoN from 7.75 ± 0.45 fold in the PTZ group to 3.20 ± 0.56, 2.67 ± 0.70, and 2.86 ± 0.26 fold at concentrations of 12, 25 and 50 μg/mL, respectively (**Figures 6D,E**).

## Discussion

Although there are long-standing controversies whether epileptic seizures lead to neuronal death, evidence has accumulated that seizures that last for a long time and occur repeatedly over a prolonged period induce neuronal death both experimentally and clinically (Liu et al., 1999; Dingledine et al., 2014; Luo et al., 2015; Naziroglu and Ovey, 2015). In this study, PTZ treatment results were consistent with this theory based on data obtained from embryonic neuronal cells, mouse cerebral cortex and zebrafish. PTZ exposure indeed increased cell death and reduced cell viability and neurite density. To date, studies on the antiepileptic effect of OtoN and its analogs are extremely limited. The only one report mainly focused on the structure analysis and indicated that otophylloside A and B could protect rats from audiogenic seizures. Thus further activities and mechanisms still remain to be explored (Mu et al., 1986).

β-Tubulin is a major microtubule element and crucial constituent of the neuronal cytoskeleton for maintaining cellular shape, surface charges and intracellular transport (Zhou and Giannakakou, 2005). Evidence has shown that even subtle changes in microtubule dynamics can impair chromosome attachment and kinetochore tension and eventually cause aberrant mitosis and cell death (Karki et al., 2013). OtoN interference preserves neuronal structure intact from PTZ toxicity in certain extent. It has been suggested that OtoN attenuates PTZ-induced neuronal injury by possibly avoiding damage to the cellular framework.

Since PARP is a substrate of caspases, the amount of cleaved PARP is a hallmark used to assess the propensity of cells to apoptosis. Meanwhile, apoptosis is regulated by several signaling pathways in which Bcl-2 protein families play key roles. Bcl-2 is an anti-apoptotic protein that acts by inhibiting apoptotic cell death, and Bax is a homolog of Bcl-2 that promotes apoptosis (Giordano et al., 2008; Sanchez et al., 2012). The Bax/Bcl-2 ratio has been shown to determine the susceptibility of cells to apoptosis. We found that OtoN treatment may protect neurons from PTZ-induced toxicity, reverse PTZ-evoked cleavage of PARP and decrease the Bax/Bcl-2 ratio. In another words, OtoN contributes to preventing PTZ-induced neuronal injury partly through inhibition of apoptosis pathway.

*c-Fos* is one of most commonly used cellular imaging among the immediate-early gene family (Bengzon et al., 2002). Its protein or mRNA products are generally used as markers indicating neuronal activation given its rapid response to stimulation (Sagar et al., 1988; Basheer et al., 1997; Cruz et al., 2015). PTZ leads to obviously upregulated *c-Fos* expression on proteins and genes. However, OtoN may decrease this trend. These results indicated an overexciting state in cortical neurons, mouse cerebral cortex and zebrafish treated with PTZ. As cellular neuronal overexciting is the basis for neuronal injury, therefore, it is reasonable to speculate that OtoN is capable of attenuate PTZ-stimulated harmful neuronal activation partially via inhibition of *c-Fos* activation.

Zebrafish have up to 80% homology with human genes, and they are accordingly an ideal model organism for use in genetics and developmental neurobiology studies (Leclercq et al., 2015; Zhang et al., 2015). An effective zebrafish model in accordance with conventional neurotoxicity assessments using histological and behavioral studies has been established to reveal promising neuroprotective drugs (Baraban et al., 2005). In our zebrafish behavioral trials, it is notable that behavior changes were completely recorded and then translated to figures by software, which implies more objective findings than simply judging the behavior changes visually. In the gene expression analysis, each mRNA sample includes at least dozens of zebrafish. As a result, the outcome itself in a certain sense reflects the overall trend of the candidate drug on zebrafish. However, compared with the cell-based *in vitro* study, OtoN has a more complex activity profile in the mice and zebrafish models, which may be partially due to various factors such as the complicated pharmacokinetic dynamics of zebrafish and mice (Afrikanova et al., 2013). In the case of the non-linear pharmacokinetics, further investigation of the concentration-dependent kinetic of OtoN remains to be conducted to help improve the dose design.

## Conclusion

We have revealed the protective effect of OtoN on primary embryonic neuronal cells. Its neuroprotective activity was further confirmed using *in vivo* mouse and zebrafish models. These results revealed that OtoN has a palpable protective effect on chemical-induced neuronal disorders, and we suggest that OtoN might be developed as a novel AED in the future.

## Author Contributions

Conceived and designed the experiments: PL and BL. Performed the experiments: FS and MC. Analyzed the data: YT, HS, CH, and JW. Contributed reagents/materials/analysis tools: CX and MZ. Wrote the paper: FS and PL.

## Conflict of Interest Statement

The authors declare that the research was conducted in the absence of any commercial or financial relationships that could be construed as a potential conflict of interest.
